# Endoscopic Management for Large Distal Urolithiasis with Horseshoe Kidney and Ureteral Crossover

**DOI:** 10.51894/001c.145817

**Published:** 2025-09-30

**Authors:** Lauren Nesi, Paramjot Gogia, Kristina D. Suson

**Affiliations:** 1 Department of Urology Detroit Medical Center, Detroit, MI, USA; 2 Department of Health Sciences Brock University, St. Catharines, ON, Canada; 3 Department of Urology Children’s Hospital of Michigan, Detroit, MI, USA

## INTRODUCTION

Horseshoe kidney (HSK) is a renal fusion anomaly most commonly characterized by functioning kidneys fused at the lower poles across the midline, forming a U-shaped organ with aberrant positioning and vasculature. HSK may be associated with complications such as vesicoureteral reflux, urinary stasis due to altered drainage, increased propensity for stone formation and hydronephrosis, and ureteropelvic junction obstruction (UPJO).

Crossed ureteral orifices, or ureteral crossover, wherein one or more ureters cross the midline before contralaterally entering the bladder, can also significantly impact urinary tract function. This may be associated with renal ectopia and can lead to stone formation and urinary tract infections (UTIs).

## CASE DESCRIPTION

We present an exceedingly rare case of a 19-year-old female with a complex medical history and concurrence of several congenital anomalies, including HSK, ureteral crossover, neurogenic bladder managed with clean intermittent catheterization, Chiari malformation, myelomeningocele, lumbar scoliosis, and imperforate anus, who developed obstructing ureteral calculi.

Initial imaging revealed two large, adjacent stones in the right distal ureter (measuring approximately 2.5cm each), complicated by HSK and ureteral crossover, imposing ureteral angulation and impacting endoscopic accessibility. The patient underwent a staged procedure with an initial stent placement and two separate ureteroscopies until she was stone-free. At first, we attempted laser lithotripsy with flexible ureteroscopy, but later opted for a semi-rigid ureteroscope due to poor visibility. This significantly reduced operative time. Despite the complexity, the patient’s stone-free status was confirmed at a follow-up ureteroscopy.

## DISCUSSION/CONCLUSIONS

The fusion of the renal poles in HSK often causes malrotation, and ureteral crossover can cause odd angles or midline kinking. Concurrently, these anomalies can complicate management and hamper endourologic access.

Endoscopic access to the distal ureter is technically challenging, potentially increasing the risk of ureteral injury. Generally, flexible ureteroscopes can better maneuver the tortuosity. In this case, however, it was preferential to safely treat large distal calculi using a semi-rigid ureteroscope. To our knowledge, it is also the first to document this concurrence of congenital anomalies in a urologic patient. Future research should explore the nuances of differential endoscopic management of urolithiasis within renal fusion and ureteral crossover anomalies.

